# Epidemiological characteristics and outcomes of COVID-19 cases: mortality inequalities by socio-economic status, Barcelona, Spain, 24 February to 4 May 2020 

**DOI:** 10.2807/1560-7917.ES.2021.26.20.2001138

**Published:** 2021-05-20

**Authors:** Julieta Politi, Mario Martín-Sánchez, Lilas Mercuriali, Blanca Borras-Bermejo, Joaquín Lopez-Contreras, Anna Vilella, Judit Villar, COVID-19 Surveillance Working Group of Barcelona, Angels Orcau, Patricia Garcia de Olalla, Cristina Rius, COVID-19 Surveillance Working Group of Barcelona, COVID-19 Surveillance Working Group of Barcelona, Anna de Andres, Dolores Alamo-Junquera, Carmen Gallego, Daniel G Abiétar, Montse Guillaumes, Joan P Millet, Emilia Molinero, Daniela Pérez León, Raquel Rodríguez, Miriam Ros, Andrés Antón, Xavier Martínez-Gómez, Tomás Pumarola, Magda Campins, Virginia Pomar, Ferran Navarro, Teresa Puig, Marta Blazquez, Inmaculada Soriano, Lourdes Barón, Clara Marín, Laura de la Torre, Xavier Castells, Margarita Posso, Juan P Horcajada, Maria A Vàrez, Laia Sentís, Miquel Gómez, Leonor Invernon, Eduardo Padilla, Mara Karaim, Noel Bordon, Francesc Fatjò, Cristina Berbel, Isabel González-Nieto, José L González, Iván Pelegrín, Eva Bargalló, Antoni Salas, Maria C Planes, Gloria García, Diego de Mendoza, Sònia Tortajada, Natàlia Juan, Jordi Casabon

**Affiliations:** 1 Epidemiology Service, Public Health Agency of Barcelona (PHAB), Barcelona, Spain; 2 Preventive Medicine and Public Health Training Unit, PSMar-UPF-PHAB (Parc de Salut Mar - Pompeu Fabra University - Public Health Agency of Barcelona), Barcelona, Spain; 3 Department of Preventive Medicine and Epidemiology, Hospital Universitari Vall d'Hebron, Universitat Autònoma de Barcelona, Barcelona, Spain; 4 Infectious Diseases-Internal Medicine, Hospital de Sant Pau-Universitat Autònoma de Barcelona, Barcelona, Spain; 5 Preventive Medicine and Epidemiology Department, Hospital Clínic, Barcelona, Spain; 6 Service of Infectious Diseases, Hospital del Mar, Barcelona, Spain; 7 Members are listed under Investigators; 8 CIBER Epidemiología y Salud Pública (CIBERESP), Barcelona, Spain; 9 Institut d’Investigació Biomèdica Sant Pau (IIB Sant Pau), Barcelona, Spain; 10 Department of Experimental and Health Sciences, Universitat Pompeu Fabra, Barcelona, Spain; 11 Department of Pediatrics, Obstetrics, Gynecology, Preventive Medicine and Public Health, Universitat Autònoma de Barcelona, Barcelona, Spain

**Keywords:** SARS-CoV-2, epidemiology, mortality, socio-economic status, health inequalities, surveillance

## Abstract

**Background:**

Population-based studies characterising outcomes of COVID-19 in European settings are limited, and effects of socio-economic status (SES) on outcomes have not been widely investigated.

**Aim:**

We describe the epidemiological characteristics of COVID-19 cases, highlighting incidence and mortality rate differences across SES during the first wave in Barcelona, Catalonia, Spain.

**Methods:**

This population-based study reports individual-level data of laboratory-confirmed COVID-19 cases diagnosed from 24 February to 4 May 2020, notified to the Public Health Agency of Barcelona and followed until 15 June 2020. We analysed end-of-study vital status and the effects of chronic conditions on mortality using logistic regression. Geocoded addresses were linked to basic health area SES data, estimated using the composed socio-economic index. We estimated age-standardised incidence, hospitalisation, and mortality rates by SES.

**Results:**

Of 15,554 COVID-19-confirmed cases, the majority were women (n = 9,028; 58%), median age was 63 years (interquartile range: 46–83), 8,046 (54%) required hospitalisation, and 2,287 (15%) cases died. Prevalence of chronic conditions varied across SES, and multiple chronic conditions increased risk of death (≥ 3, adjusted odds ratio: 2.3). Age-standardised rates (incidence, hospitalisation, mortality) were highest in the most deprived SES quartile (incidence: 1,011 (95% confidence interval (CI): 975–1,047); hospitalisation: 619 (95% CI: 591–648); mortality: 150 (95% CI: 136–165)) and lowest in the most affluent (incidence: 784 (95% CI: 759–809); hospitalisation: 400 (95% CI: 382–418); mortality: 121 (95% CI: 112–131)).

**Conclusions:**

COVID-19 outcomes varied markedly across SES, underscoring the need to implement effective preventive strategies for vulnerable populations.

## Introduction

Coronavirus disease (COVID-19) surveillance in Spain was implemented on 20 January 2020 to identify early cases and minimise onward transmission. The first confirmed case in Spain was reported in La Gomera (Canary Islands) on 31 January 2020 [1]. Recent estimates suggest that community transmission was ongoing before March throughout Spain [2,3], although this was not evident until mid-March. In response, on 14 March, the Spanish government declared a strict lockdown, which included social distancing measures, home confinement, banning of social gatherings, school closures, and restriction on civilian mobility, among other measures [4]. On 4 May, the government eased these restrictions following a sustained decrease in COVID-19 cases throughout Spain [5]. From 5 February 2020, numerous suspected COVID-19 cases were notified in Barcelona. The first confirmed COVID-19 case in the city of Barcelona occurred on 24 February 2020. In the following 10 weeks, 15,554 cases were confirmed.

Europe, which is characterised by an aging population, was strongly affected by the COVID-19 pandemic, despite well-established health systems with high coverage [6]. During the start of the epidemic, Spain was one of the most affected countries worldwide in terms of COVID-19 incidence and mortality rates [7]. Characterising and contextualising these figures is relevant to modify the course of the epidemic. In addition to describing the magnitude and severity of the crisis, regional population-wide surveillance provides a unique opportunity to characterise disease epidemiology thoroughly. In this regard, monitoring whether the COVID-19 pandemic exacerbates pre-existing health inequalities is a relevant perspective within any context to inform rapid policy responses that ensure health equity. However, data on the influence of socio-economic status (SES) and other social determinants on COVID-19 susceptibility and severity are scarce, limiting the capacity of identifying disadvantaged populations [8]. Geographically aggregated data offers valuable information for studying health inequalities by accounting for the contextual effect of the area of residence [9]. This approach is especially relevant in urban areas where health inequalities tend to be more marked [10].

The purpose of this study was to describe the epidemiological characteristics and outcomes of the first consecutive 15,554 laboratory-confirmed COVID-19 cases, as well as the cumulative incidence and mortality rate differences across SES during the first wave of COVID-19 in Barcelona, Catalonia, Spain from 24 February to 4 May 2020.

## Methods

### Design

This population-based study reports RT-PCR-confirmed COVID-19 cases notified to the Agència de Salut Pública de Barcelona (Public Health Agency of Barcelona, PHAB) and diagnosed in the city of Barcelona from 24 February to 4 May 2020.

We included all cases diagnosed up to 4 May 2020, coinciding with the easing of lockdown and restriction measures in Barcelona and the sustained decrease in COVID-19 cases within the city [5].

### Surveillance strategy

Epidemiological surveillance and control of notifiable infectious diseases is a well-established PHAB-based system in Barcelona, which is responsible for monitoring and controlling communicable disease among ca 1.6 million residents. Surveillance relies on active case finding at hospitals, primary healthcare centres, nursing homes, laboratories, and private practices. Prompt notification of suspected and confirmed cases to PHAB is required. The PHAB is part of the Catalonian surveillance network, which is operated by the Catalonia health department. 

In response to the public health emergency of international concern declared by the World Health Organisation (WHO) on 30 January 2020 and in accordance with the guidelines of the Catalonia health department, the PHAB strengthened surveillance of respiratory diseases [11]. The COVID-19 case definition was adapted from that published by the ECDC; a suspected case was defined as any individual presenting with an acute respiratory disease with at least one of the following symptoms: cough, fever or shortness of breath, and a recent travel history to an affected area or being a close contact of a confirmed or probable case [12]. Initially, affected areas included China, Japan, South Korea, Singapore, and Iran [13]. The affected northern regions of Italy (Lombardy, Emilia Romagna, Veneto, and Piedmont) were added in late February 2020. By early March 2020, the suspected case definition evolved to include severe pneumonia cases in hospitalised patients in whom no other aetiology could be determined (a minimal screening including at least influenza viruses was required to be negative). Case definitions evolved rapidly over the following days driven by test shortages, prioritising laboratory confirmation for hospitalised cases and healthcare workers while supplies were scarce, until early April.

### Laboratory analysis

As recommended by the WHO, suspected cases were tested with two real-time RT-PCR assays targeting different sequences of severe acute respiratory syndrome coronavirus 2 (SARS-CoV-2) [14]. Individuals were considered a confirmed case if both test results were positive. Since 2 April, following the Catalonia health department protocol, one positive SARS-CoV-2 RT-PCR result was sufficient for confirmation [15].

### Data sources

Data for this study were extracted from the Mandatory Registry for Infectious Diseases of the city of Barcelona. Following a case notification, the PHAB contacted each case, and trained public health professionals administered an epidemiological questionnaire that was entered into the Registry. Case records included demographic and clinical information, such as date of birth, sex, occupation, address, date of symptom onset, date of laboratory diagnosis, presenting symptoms, chronic conditions, travel history, contact with a confirmed case, hospitalisation requirements, intensive care unit (ICU) stays, and death. We defined healthcare workers (HCW) as individuals who work in a health facility. The PHAB monitored cases’ contacts, and appropriate health recommendations were provided for both cases and contacts. Public health nurses followed cases until their resolution.

Of the symptoms and clinical presentation registered, severe forms of illness, defined as pneumonia (clinical or radiological), dyspnoea, acute respiratory distress syndrome (ARDS), and acute kidney injury (AKI) are presented here. Other forms, categorised as mild, are described in the Supplement (fever, cough, diarrhoea, chills, sore throat, headache, vomiting, weakness, myalgia, anosmia, and dysgeusia). Chronic conditions were reported for diabetes, cardiovascular disease (including hypertension), chronic liver disease, chronic respiratory disease, chronic kidney disease, neurologic disease, and cancer.

Outcomes included hospitalisation, ICU stay during hospitalisation, and end-of-study vital status (alive/deceased). Deceased status was informed through hospitals, the city's funeral homes, and the central registry of insured persons (RCA: a registry recording all individuals insured through the universal public healthcare system). Cases’ vital status and information was updated until 15 June 2020, to allow a minimum follow-up of 6 weeks.

Individual SES was estimated using the composed socio-economic index, an index used for allocating resources to the Catalonian primary healthcare system since 2017 [16]. The index estimates deprivation by basic health area (BHA), by considering the following parameters: exemption from pharmaceutical co-payments, income below EUR 18,000, income above EUR 100,000, manual employment, insufficient schooling (population > 16–74 years unable to read or write, or has only primary education), premature mortality (< 75 years), and potentially avoidable hospitalisations. The index, scaled to the Catalonian population, defines a BHA deprivation range between 0 (lowest) and 100 (highest). We geocoded the address for each case and assigned the corresponding socio-economic index of the given BHA for this study. Addresses were unavailable for 294 cases. We used quartile cut-points based on the index’s distribution within Barcelona to define four SES categories: low (most deprived), medium-low, medium-high, high (most affluent).

### Analysis

Descriptive analyses were conducted to summarise key variables. Epidemiological characteristics were compared by case status at the end of the study period (alive/deceased) using the chi-squared, Wilcoxon rank-sum, or Kruskal–Wallis test, as appropriate. A p value < 0.05 was used to determine statistical significance in all analyses. A subset analysis was performed for nursing home residents. The date of laboratory confirmation was used to plot the epidemic curve. When the date of laboratory confirmation was unknown, the notification date was used to plot the epidemic curve. 

We calculated crude and standardised cumulative incidence and mortality rates per 100,000 inhabitants, stratified by SES and sex. For individuals aged 18 years and older, we investigated the relationship between chronic conditions and death using logistic regression models (crude and adjusted). Individuals younger than 18 years of age were excluded from this analysis, given their low risk of death and low prevalence of chronic conditions. We obtained adjusted odds ratios (aOR) for the risk of death by adjusting each chronic condition for age and sex. Crude (age-specific) rates were obtained by dividing the number of cases in each stratum by the corresponding stratum population size. We used a direct standardised method to calculate standardised cumulative incidence and mortality rates, using Barcelona’s total population as the reference. Population estimates were based on the RCA (as of 1 Jan 2020). We calculated the case fatality rate (CFR) as the number of deaths within a category divided by each category’s number of cases. All statistical analyses were conducted using Stata (version 15, StataCorp, College Station, Texas, United States (US)) and R software version 3.5.2 (R Foundation, Vienna, Austria).

### Ethical statement

Patients were not directly involved in this study. Only data extracted from notifiable disease surveillance systems were used. All identifiable personal information was removed for privacy protection, and therefore no informed consent was required. Data confidentiality and other ethical considerations were handled according to the international recommendations about epidemiological studies mentioned in the International Guidelines for Ethical Review of Epidemiological Studies (Council for the International 12 Organizations of Medical Sciences –CIOMS, Geneve, 1991), the Helsinki Declaration revised by the World Medical Organisation in Fortaleza in 2013, the General Data Protection Regulation (GPDR) EU 2016/679, and the Spanish Law 03/2018 on Data Protection. This study was approved by the ethics committee of the Parc de Salut Mar (CEIC-Parc de Salut MAR, registration number: 2020/9356), Barcelona.

## Results

### Epidemiological characteristics

Overall, 25,381 COVID-19 notifications were compiled. A total of 15,554 cases were laboratory confirmed and 7,939 cases (31.3%) were discarded. The remaining 1,888 cases were non-laboratory confirmed, and classified as epidemiologically-confirmed (compatible symptoms and known contact with a case, but no confirmatory testing) in 873 (3.4%), chest X-ray compatible with viral pneumonia (without laboratory confirmation) in 373 (1.5%) cases, and possible cases (consistent symptoms, without known exposures and no confirmatory testing) in 642 (2.5%).

The epidemic curve, based on the date of laboratory confirmation, is shown in the Figure. The first confirmed case in Barcelona was diagnosed on 24 February 2020. Most cases occurred between 15 March and 24 April, with one peak on 27 March and a second peak on 17 April. The median time between symptom onset and date of diagnosis varied before and after the strict lockdown decree on 14 March: the initial delay was 3 days, which increased over time reaching a median of 7 days [4]. A week after the initial case (3 March 2020), all confirmed cases (n = 16) reported either recent travel to an area with widespread transmission (eight cases reported travel to Italy) or contact with a confirmed imported case. Following this period, 4,495 (28.9%) cases reported close contact with a case, while the exposure was unknown for the remaining cases. Recent travel history was reported by 71 cases, with Italy being the most frequent destination (31.0%).

**Figure:**
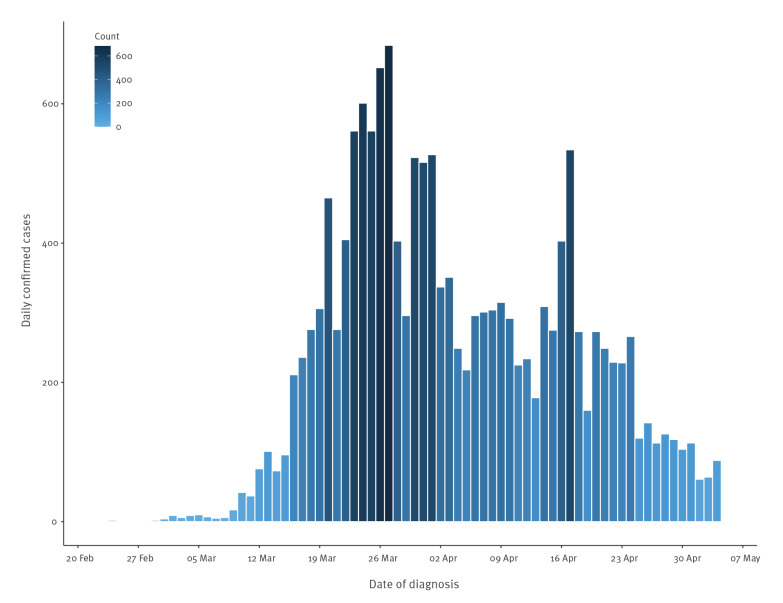
Epidemic curve of laboratory-confirmed COVID-19 cases notified to the Public Health Agency of Barcelona, Spain, 24 February–4 May 2020 (n = 15,554)

COVID-19: coronavirus disease.

### Clinical presentation and outcomes

Of the 15,554 confirmed cases, 9,028 (58.0%) were women, and the median age was 63 years (interquartile range (IQR): 46–83) (Table 1). Almost half of the cases (49.4%) were older than 65 years. Only 44 (0.3%) cases occurred in children 14 years or younger, of whom 12 were under 1 year of age. A total of 8,406 (54.0%) cases required hospitalisation, and 859 cases (5.5%) required ICU admission. Death occurred in 2,287 cases (14.7%). Information on symptoms and past medical history were available in 11,210 cases. At presentation, 5,482 (48.9%) cases had pneumonia and 5,243 (46.8%) reported dyspnoea. These proportions were significantly higher among cases with a fatal outcome than those surviving at the end of the study (p value < 0.001). The prevalence of ARDS and AKI was less frequent, although more than 30% of cases were fatal. HCW cases totalled 2,558 (16.4%).

**Table 1:** Characteristics by end-of-study status in laboratory-confirmed COVID-19 cases notified to the Public Health Agency of Barcelona, Spain, 24 February–4 May 2020 (n = 15,554)

End-of-study status	Overall n = 15,554		Alive n = 13,267		Deceased n = 2,287		p value
	n	%	n	%	n	%	
Age (median (IQR))	63 (46–83)		59 (43–78)		85 (77–90)		< 0.001
Age (years)^a^							< 0.001
0–14	44	0.3	44	0.3	0	0.0	
15–44	3,372	21.7	3,361	25.3	11	0.5	
45–64	4,443	28.6	4,334	32.7	109	4.8	
65–74	1,861	12.0	1,571	11.9	290	12.7	
75–84	2,421	15.6	1,723	13.0	698	30.5	
≥ 85	3,404	21.9	2,225	16.8	1,179	51.6	
Sex							< 0.001
Female	9,028	58.0	7,863	59.3	1,165	50.9	
Healthcare workers	2,558	16.4	2,540	19.1	18	0.8	< 0.001
Hospitalised	8,406	54.0	6,610	49.8	1,796	78.5	< 0.001
ICU	859	5.5	623	4.7	236	10.3	< 0.001
Symptoms^b^							
Pneumonia	5,482	48.9	4,453	46.8	1,029	60.7	< 0.001
Dyspnoea	5,243	46.8	4,141	43.5	1,102	65.0	< 0.001
ARDS	596	5.3	379	4.0	217	12.8	< 0.001
AKI	368	3.3	209	2.2	159	9.4	< 0.001
Chronic conditions^b^							
Cardiovascular	4,010	35.8	2,855	30.0	1,155	68.1	< 0.001
Diabetes	1,707	15.2	1,222	12.8	485	28.6	< 0.001
Respiratory	1,473	13.1	1,103	11.6	370	21.8	< 0.001
Neurologic	1,297	11.6	863	9.1	434	25.6	< 0.001
Kidney disease	905	8.1	547	5.8	358	21.1	< 0.001
Cancer	870	7.8	605	6.4	265	15.6	< 0.001
Liver disease	316	2.8	235	2.5	81	4.8	< 0.001
SES^c^							0.033
Low	3,029	19.9	2,611	20.1	418	18.3	
Medium-low	3,973	26.0	3,393	26.1	580	25.4	
Medium-high	4,478	29.3	3,809	29.4	669	29.3	
High	3,778	24.8	3,163	24.4	615	27.0	
Days from symptom onset to diagnosis (median (IQR))	7 (3–11)		7 (3–11)		5 (2–9)		< 0.001

AKI: acute kidney injury; ARDS: acute respiratory distress syndrome; COVID-19: coronavirus disease; ICU: intensive care unit upon admission; IQR: interquartile range; SES: socio-economic status.

^a^ Age was unavailable for nine cases.

^b^ Multiple symptoms and/or chronic conditions can exist for each case.

^c^ SES was unavailable for 296 cases.

Values are n (%) unless otherwise specified. Percentages for symptoms and chronic conditions are calculated based on the number of surveyed cases (n = 11,210; 9,514 alive and 1,696 deceased).

The median time between symptom onset and diagnosis was 7 days (IQR: 3–11). This interval was shorter for individuals who died by the end of the study (5 days; IQR: 2–9) (Table 1). Among HCW, the median time from symptom onset to diagnosis was 5 days (IQR: 2–9). The median time between symptom onset to diagnosis was significantly lower for the most favourable SES group, which took on average a day less than the lower three quartiles (6 days; IQR: 2–10; p = 0.0001).

### Nursing home residents

A total of 3,137 (20.2%) cases were nursing home residents (Table 2). The median age was 88 years (IQR: 83–92), and 74.1% were women. Hospitalisation was required in 33.9% of residents, and only 33 (1.1%) were admitted to the ICU. The CFR among nursing home residents was 25.6%. The most frequent presentations among residents were dyspnoea and pneumonia (54.3% and 46.0%, respectively). Chronic conditions were highly prevalent, with cardiovascular disease present in 56.8%, followed by neurological disease in 39.0% of cases. The median time between symptom onset and diagnosis was 4 days (IQR: 1–9) and 3 days (IQR: 1–8) for those who died.

**Table 2:** Characteristics by end-of-study status in laboratory-confirmed COVID-19 cases in nursing home residents in the city of Barcelona, 24 February–4 May 2020 (n = 3,137)

End-of-study status	Overall n = 3,137		Alive n = 2,334		Deceased n = 803		p value
	n	%	n	%	n	%	
Age (median (IQR))	88 (83–92)		88 (82–92)		88 (84–93)		< 0.001
Sex							< 0.001
Female	2,323	74.1	1,801	77.2	522	65.0	
Hospitalised	1,064	33.9	561	24.0	503	62.6	< 0.001
ICU	33	1.1	16	0.7	17	2.1	< 0.001
Symptoms^a^							
Pneumonia	581	46.0	300	40.2	281	54.4	< 0.001
Dyspnoea	686	54.3	343	46.0	343	66.3	< 0.001
ARDS	116	9.2	45	6.0	71	13.7	< 0.001
AKI	79	6.3	39	5.2	40	7.7	< 0.001
Chronic conditions^a^							
Cardiovascular	718	56.8	397	53.2	321	62.1	< 0.001
Diabetes	297	23.5	168	22.5	129	25.0	< 0.001
Respiratory	207	16.4	124	16.6	83	16.1	< 0.001
Neurological	493	39.0	266	35.7	227	43.9	< 0.001
Kidney disease	236	18.7	128	17.2	108	20.9	< 0.001
Cancer	113	8.9	58	7.8	55	10.6	< 0.001
Liver disease	39	3.1	22	2.9	17	3.3	< 0.001
SES							0.035
Low	395	12.6	295	12.6	100	12.5	
Medium-low	928	29.6	712	30.5	216	26.9	
Medium-high	941	30.0	695	29.8	246	30.6	
High	873	27.8	632	27.1	241	30.0	
Days from symptom onset to diagnosis (median (IQR))	4 (1–9)		5 (2–11)		3 (1–8)		< 0.001

AKI: acute kidney injury; ARDS: acute respiratory distress syndrome; COVID-19: coronavirus disease; ICU: intensive care unit upon admission; IQR: interquartile range; SES: socio-economic status; NA: not applicable.

^a^ Multiple symptoms and/or chronic conditions can exist for each case.

Values are n (%) unless otherwise specified. Percentages for symptoms and chronic conditions are calculated based on the number of surveyed cases (n = 1,263; 746 alive and 517 deceased).

### Chronic conditions

For individuals aged 18 years and above, chronic conditions were absent in 5,143 (46.1%) cases (Table 3). The risk of death increased as the number of pre-existing conditions increased, reaching an aOR of 2.3 (95% confidence interval (CI): 1.9–2.8) when three or more chronic conditions were present. Chronic conditions were more prevalent among fatal cases than those who remained alive, of which cardiovascular disease (including hypertension) was the most frequent, followed by diabetes. The prevalence of chronic conditions and their associated risk of death varied across SES, with a clear gradient for cardiovascular disease.

**Table 3:** Prevalence of chronic conditions and associated risk of death for COVID-19 cases aged 18 years and above, stratified by socio-economic status, Barcelona, Spain, 24 February–4 May 2020 (n = 11,165)

	Total	Prevalence (%)	95% CI	Deceased	aOR	95% CI	p value
Overall							
Cardiovascular	3,707	33.2	32.3–34.1	1,102	1.4	1.2–1.6	< 0.001
Diabetes	1,583	14.2	13.5–14.8	460	1.3	1.1–1.5	0.001
Respiratory	1,378	12.3	11.7–13.0	358	1.3	1.1–1.5	0.001
Neurological	1,087	9.7	9.2–10.3	403	1.5	1.3–1.8	< 0.001
Kidney disease	822	7.4	6.9–7.9	336	1.7	1.4–2.0	< 0.001
Cancer	781	7.0	6.5–7.5	243	1.5	1.3–1.8	< 0.001
Liver disease	296	2.7	2.4–3.0	78	1.5	1.1–2.0	0.008
Chronic conditions (number)							
0	5,143	46.1	45.1–47.0	240	Ref.		
1	2,537	22.7	22.0–23.5	389	1.4	1.1–1.7	0.001
2	1,968	17.6	16.9–18.3	524	1.9	1.6–2.3	< 0.001
≥ 3	1,517	13.6	13.0–14.2	543	2.3	1.9–2.8	< 0.001
SES low (n = 2,300)							
Cardiovascular	847	36.8	34.9–38.8	233	1.7	1.2–2.3	0.001
Diabetes	417	18.1	16.6–19.7	111	1.4	1.0–1.8	0.036
Respiratory	333	14.5	13.0–15.9	88	1.6	1.2–2.2	0.003
Neurological	175	7.6	6.5–8.7	49	1.2	0.8–1.7	0.454
Kidney disease	176	7.7	6.6–8.7	58	1.3	0.9–1.9	0.151
Cancer	127	5.5	4.6–6.5	44	2.0	1.3–3.1	0.002
Liver disease	90	3.9	3.1–4.7	27	2.2	1.3–3.7	0.005
SES medium-low (n = 2,763)							
Cardiovascular	927	33.6	31.8–35.3	284	1.5	1.2–1.9	0.002
Diabetes	418	15.1	13.8–16.5	121	1.2	0.9–1.5	0.303
Respiratory	346	12.5	11.3–13.8	97	1.5	1.1–2.0	0.015
Neurological	259	9.4	8.3–10.5	95	1.4	1.1- 1.9	0.024
Kidney disease	198	7.2	6.2–8.1	82	1.7	1.2–2.3	0.003
Cancer	201	7.3	6.3–8.2	56	1.3	0.9–1.8	0.223
Liver disease	62	2.2	1.7–2.8	14	1.2	0.6–2.4	0.591
SES medium-high (n = 3,225)							
Cardiovascular	1,034	32.1	30.5–33.7	304	1.4	1.1–1.7	0.008
Diabetes	386	12.0	10.9–13.1	110	1.2	0.9–1.6	0.193
Respiratory	419	13.0	11.8–14.2	101	1.1	0.8–1.4	0.551
Neurological	335	10.4	9.3–11.4	133	1.9	1.4–2.5	< 0.001
Kidney disease	228	7.1	6.2–8.0	101	2.0	1.5–2.8	< 0.001
Cancer	250	7.8	6.8–8.7	84	1.8	1.3–2.5	< 0.001
Liver disease	82	2.5	2.0–3.1	23	1.7	1.0–2.9	0.073
SES high (n = 2,720)							
Cardiovascular	866	31.8	30.1–33.6	280	1.3	1.0–1.6	0.046
Diabetes	350	12.9	11.6–14.1	118	1.4	1.1–1.9	0.009
Respiratory	269	9.9	8.8–11.0	72	1.2	0.8–1.6	0.358
Neurological	317	11.7	10.5–12.9	126	1.5	1.1–2.0	0.006
Kidney disease	216	7.9	6.9–9.0	94	1.6	1.1–2.1	0.006
Cancer	197	7.2	6.3–8.2	58	1.2	0.9–1.8	0.272
Liver disease	62	2.3	1.7–2.8	14	0.9	0.5–1.8	0.804

aOR: adjusted odds ratio by age and sex; CI: confidence intervals; COVID-19: coronavirus disease; Ref.: reference; SES: socio-economic status.

This analysis included 11,165 cases (15,496 cases were > 18 years, of whom 4,331 were excluded since they were not surveyed for chronic conditions). Independent logistic regression models were fitted for each chronic condition (adjusted by age and sex). For each model, the reference category was the absence of the condition being analysed. SES was missing in 157 cases (n = 11,008). Multiple chronic conditions can exist for each case.

### Case fatality rate

A total of 2,287 cases died by the end of the study period, with an overall CFR of 14.7%. The CFR was highest in individuals 65 years and older (28.2%). Among the deceased, 1,796 (78.5%) required hospitalisation and 236 (10.3%) ICU admission. The CFR among hospitalised cases was 21.4% and 27.5% among cases admitted to ICU (Table 1). Differences in CFR across SES were not statistically significant: the CFR was 13.8% (95% CI: 12.4–15.2) for low SES, 14.6% (95% CI: 13.3–15.9) for medium-low SES, 14.9% (95% CI: 13.7–16.2) for medium-high SES, and 16.3% (95% CI: 14.9–17.7) for high SES.

### Incidence and mortality estimates

Cumulative incidence, hospitalisation, and mortality rates per 100,000 habitants are shown in Table 4. Overall cumulative incidence rate was 946.8 (95% CI: 932.0–961.5). The overall cumulative mortality rate was 139.3 (95% CI: 133.6–145.0). While cumulative incidence rate was higher in female cases, the cumulative mortality rate was significantly higher in male cases. Age-specific incidence and mortality rates increased with age. Age-standardised cumulative incidence rates across SES showed a clear gradient, with the highest observed incidence rates in the group with lowest SES (1,010.9; 95% CI: 975.1–1,046.7). The age-standardised cumulative mortality rate across SES also revealed a gradient, with the highest mortality rate observed in the lowest SES quartile, which was significantly higher when compared with the mortality rate observed of the most affluent strata, 150.2 (95% CI: 135.9–164.6) and 121.2 (95% CI: 111.6–130.7), respectively. Cumulative age-specific incidence and mortality rates by sex are shown in the Supplement, which display similar gradients by SES in male and female cases.

**Table 4:** Cumulative COVID-19 incidence and mortality rates by sex and socio-economic status in the city of Barcelona, Spain, 24 February–4 May 2020 (n = 15,545)

Confirmed cases		Standardised cumulative incidence/100,000 (95% CI)	Hospitalisations	Standardised hospitalisation rate/100,000 (95% CI)	Deaths	Standardised cumulative mortality/100,000 (95% CI)
Barcelona^a^	15,545	946.8 (932.0–961.5)	8,404	511.8 (501.0–522.7)	2,287	139.3 (133.6–145.0)
Sex						
Female	9,023	968.9 (948.9–988.9)	4,091	434.2 (420.9–447.58)	1,165	110.4 (104.1–116.8)
Male	6,522	927.9 (905.4–950.5)	4,313	621.2 (602.5–639.8)	1,122	185.4 (174.0–196.0)
SES^b^						
Low	3,029	1,010.9 (975.1–1,046.7)	1,837	619.4 (591.1–647.6)	418	150.2 (135.9–164.6)
Medium-low	3,973	993.0 (962.5–1,023.6)	2,069	517.7 (495.5–539.9)	580	145.6 (133.8–157.4)
Medium-high	4,478	968.0 (940.0–996.1)	2,417	522.3 (501.6–543)	669	145.3 (134.4–156.3)
High	3,778	783.9 (759.1–808.9)	1,955	400.0 (382.3–417.7)	615	121.2 (111.6–130.7)

CI: confidence intervals; COVID-19: coronavirus disease; SES: socio-economic status.

^a^ Measures for Barcelona are unadjusted, given that these represent population totals.

^b^ SES was unavailable for 287 cases, of which 126 were hospitalisations and five were deaths.

Of the 15,554 cases in the study period, nine were excluded due to missing age. Population data obtained from the central registry of insured persons (as of 1 Jan 2020). Standardised mortality and hospitalisation rates by sex were calculated using Barcelona's population as reference.

## Discussion

We report results from a large population-wide cohort of confirmed COVID-19 cases in Spain during the first wave of the pandemic. The start of the epidemic (February to May 2020) in the city of Barcelona exemplifies a rapidly evolving situation, consistent with the ongoing COVID-19 pandemic [17]. The epidemic curve fits with a propagated epidemic pattern, with successively larger peaks with each incubation period, and a peak at 4 weeks after the first confirmed case. Case distribution by age, presenting symptoms, and chronic conditions was similar to previous reports from China and the US [18,19]. In our study, hospitalisation was required frequently (54.0%), and the CFR among hospitalised cases was 21.4%. Cumulative incidence rates were higher in women and with increasing age. Conversely, as previously reported in other studies, the cumulative mortality rate was higher in males and individuals 65 years and older [18]. Cumulative incidence and mortality rates varied across SES, with a clear gradient demonstrating higher incidence and mortality rates in those most deprived.

Our study was based on COVID-19 laboratory-confirmed cases, and mortality rates reflect deaths occurring in this group. Since testing remained available for cases seeking hospital care throughout the entire study period, we consider that our results best describe moderate and severe COVID-19 cases. Therefore, CFR should be interpreted with caution, as with any ongoing epidemic, because the denominator remains unknown (given limited testing capacity and an unknown count of mild or asymptomatic cases). However, if we consider the results from the first round of the national seroprevalence study, which was carried out at the end of our study period, the estimated seroprevalence for Barcelona was at 7.1% [20]. Considering Barcelona’s population (1.6 million), the total number of infections would total 113,600. Following these assumptions and expecting an accurate number of deaths, a rough population-wide CFR estimate could approximate 2.01%, similar to those reported for Wuhan (2.2%) or China (2.3%) [21,22]. Our surveillance system may offer more reliable estimates of hospital CFR than previous reports due to greater efficiency in capturing deaths occurring outside hospital settings (such as nursing homes or personal residencies) [18,23]. We report a CFR for hospitalised patients of 21.4%, which is lower than values reported for China, while similar to those reported in the US [18,23]. While these findings are surprising, given the differences in population and healthcare characteristics between Spain and these two countries, the nature of our data (population-wide vs hospital case series) may explain why our hospital CFR does not differ from those in the US. A relative preparedness gained from China’s experience could explain the hospital-based CFR were lower in our setting.

SES has long been recognised as a determinant in the incidence of infectious diseases. Previous studies suggest that pandemic outcomes are influenced by SES [24-26]. Despite this, a recent review of international pandemic preparedness plans highlighted the lack of consideration given to social inequalities [27]. We report a clear incidence and mortality rate gradient between different SES within our city. For instance, mortality rate spanned from 121.2 per 100,000 inhabitants (95% CI: 112–131) in the most affluent SES quartile to 150.2 (95% CI: 136–165) in the lowest and most deprived SES quartile. We hypothesise that several mechanisms could explain our findings. Firstly, quarantine measures may increase health inequalities, especially among the disadvantaged. In this sense, increased exposure between crowded household members is highly possible and probably coupled with other factors including low home quality, and lack of ventilation or proper disinfection measures. As with influenza, household crowding, along with SES and poverty, have been positively associated with hospitalisation rates [25,26,28,29]. Furthermore, most cluster transmission reported in China occurred within family settings, which is likely to reflect our setting of large extended families residing together in crowded homes [30]. Secondly, individuals in more disadvantaged SES may be more dependent on public transportation, or employed in essential jobs for which working from home is not possible. Thirdly, as we and others have shown, the COVID-19 mortality rate is higher among those with underlying chronic conditions [18,31]. Disadvantaged SES has consistently been associated with a higher prevalence of chronic conditions such as heart disease, obesity, and diabetes [32,33]. We expected a higher CFR with lower SES, given the higher prevalence of chronic conditions among people of lower SES. However, this was not reflected in our data, where no significant differences were observed across SES. While many factors could explain this finding, such as testing restrictions for people without a severe presentation (overestimating the real CFR) or barriers in access to care, an alternative could be that CFR among severe cases is similar across SES strata in a well-developed universal healthcare system. However, our data fail to completely explain the underlying relationship between SES, chronic conditions, and COVID-19 incidence and mortality rates. Higher disease incidence and mortality rates could be explained both by higher exposure and higher susceptibility to disease. More sophisticated analyses, beyond the scope of this study, are needed to better understand these relationships. Despite this, our results emphasise the need to act on relevant determinants of health [34].

We observed a mortality rate gradient across low and medium SES in those older than 65 years but differences were not significant. This is relevant because most nursing homes in Barcelona lie within a medium SES BHA, which could have blurred a steeper gradient in mortality rates between these quartiles. To what extent a nursing home's location represents an individual’s SES is a valid question. Interestingly, studies focusing on nursing home inequalities have associated lower quality of care with both nursing home residents’ SES and the SES of nursing home's location [35]. Widespread testing was not available for nursing homes until early April 2020. Therefore, cases and mortality rates may be under-reported. Excess mortality methods may be more appropriate to evaluate further differences [36-38]. The CFR for this population was considerable, at 26%, although this is in range with other reports [39]. Our estimates underscore the need to implement effective surveillance in these settings, as well as preventive strategies for vulnerable populations, especially for elderly people and other deprived groups [40]. Further studies comparing different nursing home settings, such as size, ownership, nurse to patient ratios, patient safety, and quality of life are relevant to implement appropriate prevention and control measures.

Many factors may have influenced the widespread transmission dynamics. On average, the PHAB receives around 14,000 mandatory notifications of infectious diseases yearly. Roughly 10 weeks after the first confirmed COVID-19 case, notifications increased rapidly, reaching a total of 25,381 notifications and 15,554 confirmed cases. This rapid rise in notifications made contact tracing difficult because of limited resources, which delayed contact identification and implementation of quarantine measures. The strict lockdown decree may have at least partially remedied this deficiency, by considerably reducing the number of contacts for each case and thereby transmission. A shortage of personal protective equipment (PPE) was prevalent throughout, and many settings lacked proper PPE training. An additional source of diagnostic lag time was the requirement of public health agents’ authorisation for all SARS-CoV-2 RT-PCR testing. Until 15 March 2020, a public health professional reviewed whether each case fulfilled epidemiological criteria before test authorisation. Following this period, and driven by test shortage, testing was reserved for HCW and hospitalised cases, and hospitals were autonomous to prioritize tests, expediting the process. Initial case definitions were highly specific, which may have caused initial cases to be missed because the testing criteria were not fulfilled. Furthermore, upon a negative RT-PCR test, cases were not required to isolate themselves. False negatives, along with asymptomatic transmission, were likely additional contributing factors in the spread of the epidemic, in a setting with significant testing constraints.

This study had some limitations. Firstly, data used for this study most probably represent laboratory-confirmed COVID-19 cases who sought care. While testing may have varied across centres, standard guidelines probably maintained uniformity across health facilities. Secondly, a deceased status only reflects deaths occurring in individuals with laboratory-confirmed COVID-19 and the direct cause of death may be of another reason. Thirdly, while SES was assessed based on BHA, this may not reflect individual SES status. While smallest available area data (census tract area) are recommended for ecological SES estimates to maintain a higher homogeneity within the area, we favoured the composed socio-economic index because it relied on recent area estimates for each BHA [16,41]. Furthermore, the index we used is reported to be consistent with Medea index estimates [16]. Fourthly, under-reporting may have been an issue, especially for ICU stays, since this information was collected primarily at admission. Finally, CFR may be especially underestimated among cases admitted to ICU, since the survival of cases that required ICU care may be more appropriately estimated at 90 days [42].

During the following waves of COVID-19 across Europe, a higher percentage of cases were younger and asymptomatic in Barcelona. However, incidence rates remain higher in the most deprived neighbourhoods [43]. At the time of revising this report, we could not compare the first to subsequent waves because of substantial changes regarding epidemiological procedures, the use of an abbreviated epidemiological survey, and the local registry's transition to a centralised regional registry.

## Conclusion

During the first wave of COVID-19 in Barcelona, outcomes varied by sex, age, chronic conditions, and SES. Our results underscore the need to implement effective preventive strategies for vulnerable populations, especially for elderly and underprivileged people. Long-term social inequities should be reduced in order to lessen health inequalities. These findings may inform public health policy in large cities of Spain, as well as other countries and regions. Rapid case diagnosis with prompt contact tracing should remain a priority to improve system efficiency. Considerable technical and human resources should be allocated to public health, without ignoring the health system and social support measures that may be required.

## Supplementary Data

283000 Supplement
