# Supplementary material for: Epidemiological characteristics and outcomes of COVID-19 cases: mortality inequalities by socio-economic status, Barcelona, Spain, 24 February to 4 May 2020
Source: Euro Surveill. 2021 May 20;26(20):2001138. doi: 10.2807/1560-7917.ES.2021.26.20.2001138 (PMC8138960; doi:10.2807/1560-7917.ES.2021.26.20.2001138)
Supplement: Supplement [file 20-01138_GARCIA-DE-OLALLA_Supplement.pdf]

Supplementary material

This supplementary material is hosted by ***Eurosurveillance*** as supporting information alongside the article ***Epidemiological Characteristics and Outcomes of COVID-19 cases in Barcelona, Spain: Mortality Inequalities by Socioeconomic Status***, on behalf of the authors, who remain responsible for the accuracy and appropriateness of the content. The same standards for ethics, copyright, attributions and permissions as for the article apply. Supplements are not edited by *Eurosurveillance* and the journal is not responsible for the maintenance of any links or email addresses provided therein.

Content:

Appendix 1. Distribution of mild symptoms among COVID-19 cases in the city of Barcelona until May 4, 2020
Appendix 2. Cumulative COVID-19 incidence and mortality in the city of Barcelona by sex, age and socioeconomic status until May 4, 2020

Appendix 1: **Distribution of mild symptoms among COVID-19 cases in the city of Barcelona until May 4, 2020**

		**End-of-study status**		
	**Overall**	**Alive**	**Deceased**	**p**
**n**	**11210**	**9514**	**1696**	
**Symptoms**				
Fever (%)	8506 (76.3)	7237 (76.4)	1269 (75.5)	0.379
Cough (%)	7259 (65.63)	6395 (68.0)	864 (52.2)	<0.0001
Weakness (%)	2875 (26.2)	2601 (27.9)	274 (16.8)	<0.0001
Diarrhea (%)	2675 (24.6)	2445 (26.3)	230 (14.11)	<0.0001
Headache (%)	2057 (18.9)	2015 (21.8)	42 (2.6)	<0.0001
Myalgia (%)	1917 (17.6)	1850 (19.9)	67 (4.1)	<0.0001
Chills (%)	1412 (13.1)	1316 (14.34)	96 (6.0)	<0.0001
Vomiting (%)	802 (7.4)	720 (7.8)	82 (5.1)	<0.0001
Sore throat (%)	1196 (11.1)	1153 (12.5)	43 (2.7)	<0.0001
Anosmia (%)	1591 (14.6)	1579 (17.0)	12 (0.7)	<0.0001
Dysgeusia	1095 (10.0)	1086 (11.7)	9 (0.6)	<0.0001
Other symptoms (%)	2871 (25.7)	2435 (25.6)	436 (25.7)	0.921

Values are n (%) unless otherwise specified. Symptoms could be multiple for each case.
Percentages are calculated over the total number of cases with symptoms surveyed (n=11210).

Appendix 2: **Cumulative COVID-19 incidence and mortality in the city of Barcelona by sex, age and socioeconomic status until May 4, 2020**

	**Population**	**Confirmed Cases**	**Cumulative Incidence per 100 000 (95%CI)**	**Standardized Cumulative Incidence per 100 000 (95%CI)**	**Hospitalizations**	**Hospitalization rate per 100 000 (95%CI)**	**Standardized Hospitalization rate per 100 000 (95%CI)**	**Deaths**	**CFR (95%CI)**	**Cumulative Mortality per 100 000 (95%CI)**	**Standardized Cumulative Mortality per 100 000 (95%CI)**
**Total**	1641875	15545	946.8 (932-961.5)	NA	8404	511.9 (501-522.7)	NA	2287	14.7 (14.1-15.4)	139.3 (133.6-145)	NA
**Age group**											
0-14	199776	44	22 (15.5-28.5)	NA	13	6.5 (3-10)	NA	0	0 (0-8.84)	0 (0-8.84)	NA
15-44	635426	3372	530.7 (512.8-548.6)	NA	1050	165.2 (155.2-175.2)	NA	11	0.3 (0.1-0.5)	1.7 (0.7-2.8)	NA
45-64	450247	4443	986.8 (957.8-1015.8)	NA	2494	553.9 (532.2-575.7)	NA	109	2.5 (2-2.9)	24.2 (19.7-28.8)	NA
65-74	167105	1861	1113.7 (1063.1-1164.3)	NA	1486	889.3 (844-934.5)	NA	290	15.6 (13.6-17.5)	173.5 (153.6-193.5)	NA
75-84	118962	2421	2035.1 (1954-2116.2)	NA	1686	1417.3 (1349.6-1484.9)	NA	698	28.8 (26.3-31.4)	586.7 (543.2-630.3)	NA
85+	70359	3404	4838 (4675.5-5000.6)	NA	1675	2380.6 (2266.6-2494.7)	NA	1179	34.6 (32.2-37.1)	1675.7 (1580-1771.3)	NA
**Sex***											
Females	864151	9023	1044.1 (1022.6-1065.7)	968.9 (948.9-988.9)*	4091	473.4 (458.9-487.9)	434.2 (420.9-447.6)*	1165	12.9 (12.1-13.7)	134.8 (127.1-142.6)	110.4 (104.1-116.7)*
Males	777724	6522	838.6 (818.1-859.0)	927.9 (905.3-950.5)*	4313	554.6 (538.0-571.2)	621.2 (602.5-639.8)*	1122	17.2 (16.1-18.3)	144.3 (135.8-152.7)	185.4 (174-196)*
**SES**											
**Low**	316376	3029	957.4 (923.3-991.5)	1010.9 (975.1-1046.7)	1837	580.6 (554.1-607.2)	619.4 (591.1-647.6)	418	13.8 (12.4-15.2)	132.1 (119.5-144.8)	150.2 (135.9-164.6)
0-14	41701	5	12 (1.5-22.5)	NA	3	7.2 (-0.9-15.3)	NA	0	0 (0-8.84)	0 (0-8.84)	NA
15-44	129160	744	576 (534.6-617.4)	NA	301	233 (206.7-259.4)	NA	3	0.4 (-0.1-0.9)	2.3 (-0.3-5)	NA
45-64	85373	948	1110.4 (1039.7-1181.1)	NA	604	707.5 (651.1-763.9)	NA	24	2.5 (1.5-3.6)	28.1 (16.9-39.4)	NA

65-74	27312	336	1230.2 (1098.7-1361.8)	NA	278	1017.9 (898.2-1137.5)	NA	56	16.7 (11.9-21.4)	205 (151.3-258.7)	NA
75-84	21624	508	2349.2 (2144.9-2553.5)	NA	385	1780.4 (1602.6-1958.3)	NA	155	30.5 (24.7-36.3)	716.8 (604-829.6)	NA
85+	11206	488	4354.8 (3968.4-4741.2)	NA	266	2373.7 (2088.5-2659)	NA	180	36.9 (30.1-43.7)	1606.3 (1371.6-1840.9)	NA
**Med-Low**	399309	3973	994.7(963.7-1025.6)	993.0 (962.5-1023.6)	2069	518.1 (495.8-540.5)	517.7 (495.5-539.9)	580	14.6 (13.3-15.9)	145.3 (133.4-157.1)	145.6 (133.8-157.4)
0-14	47195	12	25.4 (11-39.8)	NA	4	8.5 (0.2-16.8)	NA	0	0 (0-8.84)	0 (0-8.84)	NA
15-44	155522	802	515.7 (480.0-551.4)	NA	245	157.5 (137.8-177.3)	NA	0	0 (0-8.84)	0 (0-8.84)	NA
45-64	110815	1107	999 (940.1-1057.8)	NA	626	564.9 (520.7-609.2)	NA	29	2.6 (1.7-3.6)	26.2 (16.6-35.7)	NA
65-74	39369	456	1158.3 (1052.0-1264.6)	NA	349	886.5 (793.5-979.5)	NA	72	15.8 (11.8-19.8)	182.9 (140.6-225.1)	NA
75-84	29427	648	2202.1 (2032.5-2371.6)	NA	435	1478.2 (1339.3-1617.2)	NA	173	26.7 (22.1-31.3)	587.9 (500.3-675.5)	NA
85+	16981	947	5576.8 (5221.6-5932.0)	NA	410	2414.5 (2180.7-2648.2)	NA	306	32.3 (27.9-36.8)	1802 (1600.1-2003.9)	NA
**Med-High**	459681	4478	974.2 (945.6-1002.7)	968 (940-996.1)	2417	525.8 (504.8-546.8)	522.3 (501.6-543)	669	14.9 (13.7-16.2)	145.5 (134.5-156.6)	145.3 (134.4-156.3)
0-14	52313	9	17.2 (6-28.4)	NA	0	0 (0-8.84)	NA	0	0 (0-8.84)	0 (0-8.84)	NA
15-44	179807	973	541.1 (507.1-575.1)	NA	293	163 (144.3-181.6)	NA	5	0.5 (0.1-1)	2.8 (0.3-5.2)	NA
45-64	126690	1275	1006.4 (951.2-1061.6)	NA	705	556.5 (515.4-597.6)	NA	34	2.7 (1.8-3.6)	26.8 (17.8-35.9)	NA
65-74	48704	569	1168.3 (1072.3-1264.3)	NA	465	954.7 (868-1041.5)	NA	88	15.5 (12-19)	180.7 (142.9-218.4)	NA
75-84	32280	629	1948.6 (1796.3-	NA	436	1350.7 (1223.9-1477.5)	NA	188	29.9 (24.8-35)	582.4 (499.2-	NA

			2100.9)							665.7)	
85+	19887	1023	5144.1 (4828.8-5459.3)	NA	518	2604.7 (2380.4-2829)	NA	354	34.6 (30.1-39.1)	1780.1 (1594.6-1965.5)	NA
**High**	466509	3778	809.8(784.0-835.6)	783.9(759.1-808.9)	1955	419.1 (400.5-437.6)	400 (382.3-417.7)	615	16.3 (14.9-17.7)	131.8 (121.4-142.2)	121.2 (111.6-130.7)
0-14	58567	13	22.2 (10.1-34.3)	NA	3	5.1 (-0.7-10.9)	NA	0	0 (0-8.84)	0 (0-8.84)	NA
15-44	170937	707	413.6 (383.1-444.1)	NA	167	97.7 (82.9-112.5)	NA	2	0.3 (-0.1-0.7)	1.2 (-0.5-2.8)	NA
45-64	127369	1049	823.6 (773.8-873.4)	NA	523	410.6 (375.4-445.8)	NA	22	2 (1.1-2.9)	17.3 (10.1-24.5)	NA
65-74	51720	479	926.1 (843.2-1009.1)	NA	376	727 (653.5-800.5)	NA	74	15.4 (11.6-19.3)	143.1 (110.5-175.7)	NA
75-84	35631	618	1734.4 (1597.7-1871.2)	NA	418	1173.1 (1060.7-1285.6)	NA	180	29.1 (24.1-34.2)	505.2 (431.4-579)	NA
85+	22285	912	4092.4 (3826.8-4358)	NA	468	2100.1 (1909.8-2290.3)	NA	337	37 (32-41.9)	1512.2 (1350.8-1673.7)	NA
**Females-SES**											
**Low**	159706	1703	1066.3 (1015.7-1161.9)	1050.9 (1001.3-1100.4)	890	557.3 (520.7-593.9)	549.9 (513.9-585.9)	208	12.2 (10.4-14)	130.2 (112.5-147.9)	121.2 (104.8-137.7)
0-14	20288	3	14.8 (-1.9-31.5)	NA	1	4.9 (4.7-14.6)	NA	0	0 (0-8.84)	0 (0-8.84)	NA
15-44	61397	446	726.4 (659-793.8)	NA	151	245.9 (206.7-285.2)	NA	1	0.2 (-0.2-0.7)	1.6 (1.6-4.8)	NA
45-64	42496	501	1178.9 (1075.7-1282.2)	NA	270	635.4 (559.6-711.1)	NA	7	1.4 (0.4-2.4)	16.5 (4.3-28.7)	NA
65-74	15166	167	1101.1 (934.1-1268.2)	NA	140	923.1 (770.2-1076)	NA	25	15 (8.6-21.3)	164.8 (100.2-229.5)	NA
75-84	12835	238	1854.3 (1618.7-2089.9)	NA	172	1340.1 (1139.8-1540.4)	NA	68	28.6 (20.5-36.6)	529.8 (403.9-655.7)	NA
85+	7524	348	4625.2 (4139.2-5111.2)	NA	156	2073.4 (1748-2398.7)	NA	107	30.7 (23.7-37.7)	1422.1 (1152.7-1691.6)	NA

**Medium-Low**	209026	2334	1116.6 (1071.3-1161.9)	1027.8 (986.2-1069.3)	997	477 (447.4-506.6)	437 (409.8-464.2)	303	13 (11.4-14.5)	145 (128.6-161.3)	118.8 (105.5-132.2)
0-14	22927	4	17.4 (0.3-34.5)	NA	1	4.4 (4.2-12.9)	NA	0	0 (0-8.84)	0 (0-8.84)	NA
15-44	77936	495	635.1 (579.2-691.1)	NA	108	138.6 (112.4-164.7)	NA	0	0 (0-8.84)	0 (0-8.84)	NA
45-64	57075	608	1065.3 (980.6-1149.9)	NA	283	495.8 (438.1-553.6)	NA	10	1.6 (0.6-2.7)	17.5 (6.7-28.4)	NA
65-74	22123	209	944.7 (816.6-1072.8)	NA	153	691.6 (582-801.2)	NA	25	12 (7-17)	113 (68.7-157.3)	NA
75-84	17348	329	1896.5 (1691.5-2101.4)	NA	200	1152.9 (993.1-1312.7)	NA	75	22.8 (16.9-28.7)	432.3 (334.5-530.2)	NA
85+	11617	689	5931 (5488.1-6373.8)	NA	252	2169.2 (1901.4-2437.1)	NA	193	28 (23.4-32.7)	1661.4 (1427-1895.7)	NA
**Medium-High**	243898	2641	1082.8 (1041.5-1124.1)	996.3 (958.4-1034.2)	1186	486.3 (458.6-513.9)	441.4 (416.2-466.6)	334	12.6 (11.2-14.1)	136.9 (122.3-151.6)	111.6 (99.7-123.6)
0-14	25378	5	19.7 (2.4-37)	NA	0	0 (0-8.84)	NA	0	0 (0-8.84)	0 (0-8.84)	NA
15-44	91350	586	641.5 (589.5-693.4)	NA	138	151.1 (125.9-176.3)	NA	2	0.3 (-0.1-0.8)	2.2 (0.8-5.2)	NA
45-64	66751	708	1060.7 (982.5-1138.8)	NA	313	468.9 (417-520.9)	NA	9	1.3 (0.4-2.1)	13.5 (4.7-22.3)	NA
65-74	27451	264	961.7 (845.7-1077.7)	NA	205	746.8 (644.6-849)	NA	32	12.1 (7.6-16.6)	116.6 (76.2-157)	NA
75-84	19170	328	1711 (1525.8-1896.2)	NA	204	1064.2 (918.1-1210.2)	NA	72	22 (16.2-27.7)	375.6 (288.8-462.3)	NA
85+	13798	750	5435.6 (5046.6-5824.6)	NA	326	2362.7 (2106.2-2619.1)	NA	219	29.2 (24.6-33.8)	1587.2 (1377-1797.4)	NA
**High**	251521	2177	865.5 (829.2-901.9)	777.4 (744.5-810.3)	953	378.9 (354.8-403)	329.2 (308.1-350.2)	318	14.6 (12.9-16.3)	126.4 (112.5-140.3)	95.9 (85.3-106.5)
0-14	28587	6	21 (4.2-37.8)	NA	2	7 (2.7-16.7)	NA	0	0 (0-8.84)	0 (0-8.84)	NA
15-44	88460	432	488.4 (442.3-	NA	79	89.3 (69.6-109)	NA	1	0.2 (-0.2-	1.1 (1.1-3.3)	NA

			534.4)						0.7)		
45-64	68402	550	804.1 (736.9-871.3)	NA	226	330.4 (287.3-373.5)	NA	4	0.7 (0-1.4)	5.8 (0.1-11.6)	NA
65-74	29211	206	705.2 (608.9-801.5)	NA	158	540.9 (456.6-625.2)	NA	27	13.1 (7.8-18.4)	92.4 (57.6-127.3)	NA
75-84	21331	350	1640.8 (1468.9-1812.7)	NA	211	989.2 (855.7-1122.6)	NA	87	24.9 (18.8-30.9)	407.9 (322.2-493.6)	NA
85+	15530	633	4076 (3758.5-4393.5)	NA	277	1783.6 (1573.6-1993.7)	NA	199	31.4 (26.2-36.7)	1281.4 (1103.4-1459.4)	NA
**Males-SES**											
Low	159706	1326	846.4 (800.8-891.9)	984.4 (930-1038)	947	604.5 (-566-643)	719.7 (673-766)	210	15.8 (13.5-18.2)	134 (115.9-152.2)	194.7 (168-221)
0-14	20288	2	9.3 (-3.6-22.3)	NA	2	9.3 (3.6-22.3)	NA	0	0 (0-8.84)	0 (0-8.84)	NA
15-44	61397	298	439.8 (389.8-489.7)	NA	150	221.4 (-185.9-256.8)	NA	2	0.7 (-0.3-1.6)	3 (-1.1-7)	NA
45-64	42496	447	1042.5 (945.9-1139.2)	NA	334	779 (-695.4-862.5)	NA	17	3.8 (2-5.6)	39.6 (20.8-58.5)	NA
65-74	15166	169	1391.4 (1181.6-1601.2)	NA	138	1136.2 (-946.6-1325.7)	NA	31	18.3 (11.2-25.5)	255.2 (165.4-345.1)	NA
75-84	12835	270	3072 (2705.6-3438.5)	NA	213	2423.5 (-2098-2749)	NA	87	32.2 (24-40.4)	989.9 (781.9-1197.9)	NA
85+	7524	140	3802.3 (3172.4-4432.1)	NA	110	2987.5 (-2429.2-3545.8)	NA	73	52.1 (34.9-69.4)	1982.6 (1527.8-2437.4)	NA
Medium-Low	209026	1639	861.3 (819.6-903)	955.5 (909-1002)	1072	563.4 (-529.6-597.1)	627.7 (590-666)	277	16.9 (14.7-19.1)	145.6 (128.4-162.7)	186.5 (164-209)
0-14	22927	8	33 (10.1-55.8)	NA	3	12.4 (1.6-26.4)	NA	0	0 (0-8.84)	0 (0-8.84)	NA
15-44	77936	307	395.7 (351.4-440)	NA	137	176.6 (-147-206.1)	NA	0	0 (0-8.84)	0 (0-8.84)	NA
45-64	57075	500	930.4 (848.9-1012)	NA	343	638.3 (-570.7-705.8)	NA	19	3.8 (2.1-5.5)	35.4 (19.5-51.3)	NA
65-74	22123	247	1432.2 (1253.6-	NA	196	1136.5 (-977.4-1295.6)	NA	47	19 (13-25.1)	272.5 (194.6-	NA

			1610.8)							350.4)	
75-84	17348	319	2640.9 (2351.1-2930.8)	NA	235	1945.5 (-1696.8-2194.3)	NA	98	30.7 (23.4-38)	811.3 (650.7-972)	NA
85+	11617	258	4809.8 (4222.9-5396.8)	NA	158	2945.6 (-2486.3-3404.9)	NA	113	43.8 (33-54.6)	2106.6 (1718.2-2495.1)	NA
Medium-High	243898	1837	851.3 (812.4-890.2)	935.1 (892-978)	1231	570.5 (538.6-602.3)	635 (599-671)	335	18.2 (16.1-20.4)	155.2 (138.6-171.9)	198.7 (177-220)
0-14	25378	4	14.9 (0.3-29.4)	NA	0	0 (0-8.84)	NA	0	0 (0-8.84)	0 (0-8.84)	NA
15-44	91350	387	437.5 (393.9-481.1)	NA	155	175.2 (147.6-202.8)	NA	3	0.8 (-0.1-1.7)	3.4 (-0.4-7.2)	NA
45-64	66751	567	946 (868.1-1023.8)	NA	392	654 (589.3-718.7)	NA	25	4.4 (2.6-6.2)	41.7 (25.4-58.1)	NA
65-74	27451	305	1435.1 (1274-1596.2)	NA	260	1223.4 (1074.7-1372.1)	NA	56	18.4 (13-23.7)	263.5 (194.5-332.5)	NA
75-84	19170	301	2296 (2036.6-2555.3)	NA	232	1769.6 (1541.9-1997.4)	NA	116	38.5 (29.6-47.5)	884.8 (723.8-1045.8)	NA
85+	13798	273	4483.5 (3951.6-5015.3)	NA	192	3153.2 (2707.2-3599.3)	NA	135	49.5 (37.7-61.2)	2217.1 (1843.1-2591.1)	NA
High	251521	1601	744.7 (708.2-781.2)	800.2 (761-839)	1002	466.1 (-437.2-494.9)	504.4 (473-536)	297	18.6 (16.2-20.9)	138.1 (122.4-153.9)	164.8 (146-184)
0-14	28587	7	23.3 (6.1-40.6)	NA	1	3.3 (3.2-9.9)	NA	0	0 (0-8.84)	0 (0-8.84)	NA
15-44	88460	275	333.4 (294-372.8)	NA	88	106.7 (-84.4-129)	NA	1	0.4 (-0.4-1.1)	1.2 (-1.2-3.6)	NA
45-64	68402	499	846.2 (772-920.5)	NA	297	503.7 (-446.4-561)	NA	18	3.6 (1.9-5.3)	30.5 (16.4-44.6)	NA
65-74	29211	273	1212.8 (1069-1356.7)	NA	218	968.5 (-839.9-1097.1)	NA	47	17.2 (11.8-22.6)	208.8 (149.1-268.5)	NA
75-84	21331	268	1874.1 (1649.7-2098.5)	NA	207	1447.6 (-1250.4-1644.8)	NA	93	34.7 (26-43.4)	650.3 (518.2-782.5)	NA
85+	15530	279	4130.3 (3645.6-4614.9)	NA	191	2827.5 (-2426.5-3228.5)	NA	138	49.5 (37.9-61.1)	2042.9 (1702.1-2383.8)	NA

Population data obtained from the central insured registry of Catalonia, as of 2020. SES was unavailable for 294 cases, of which 6 were deaths. Standardized mortality and hospitalization rates for age and sex were calculated using Barcelona's population as reference. *Sex was standardized for age only. 95%CI=95% confidence intervals; CFR=Case fatality rate: NA=Not Applicable; SES=socioeconomic status; Med-Low=Medium-low category; Med-High=Medium-high category.
